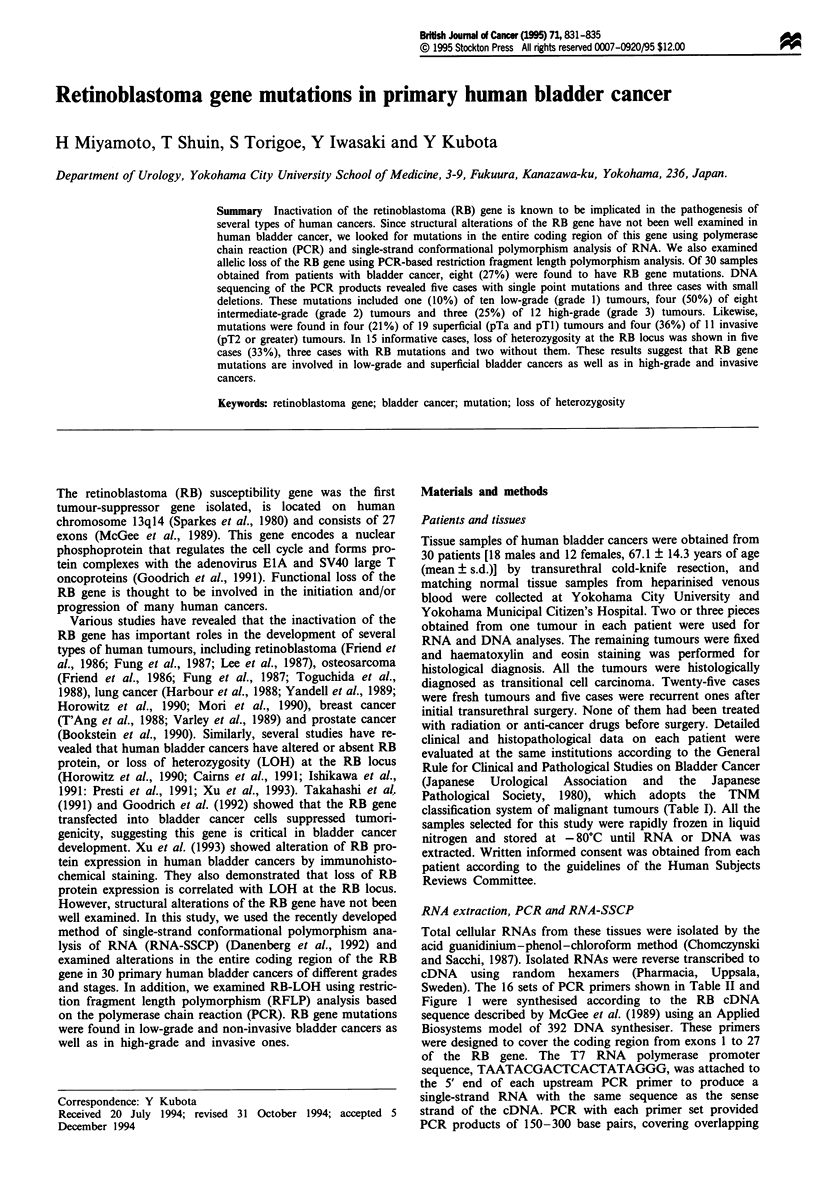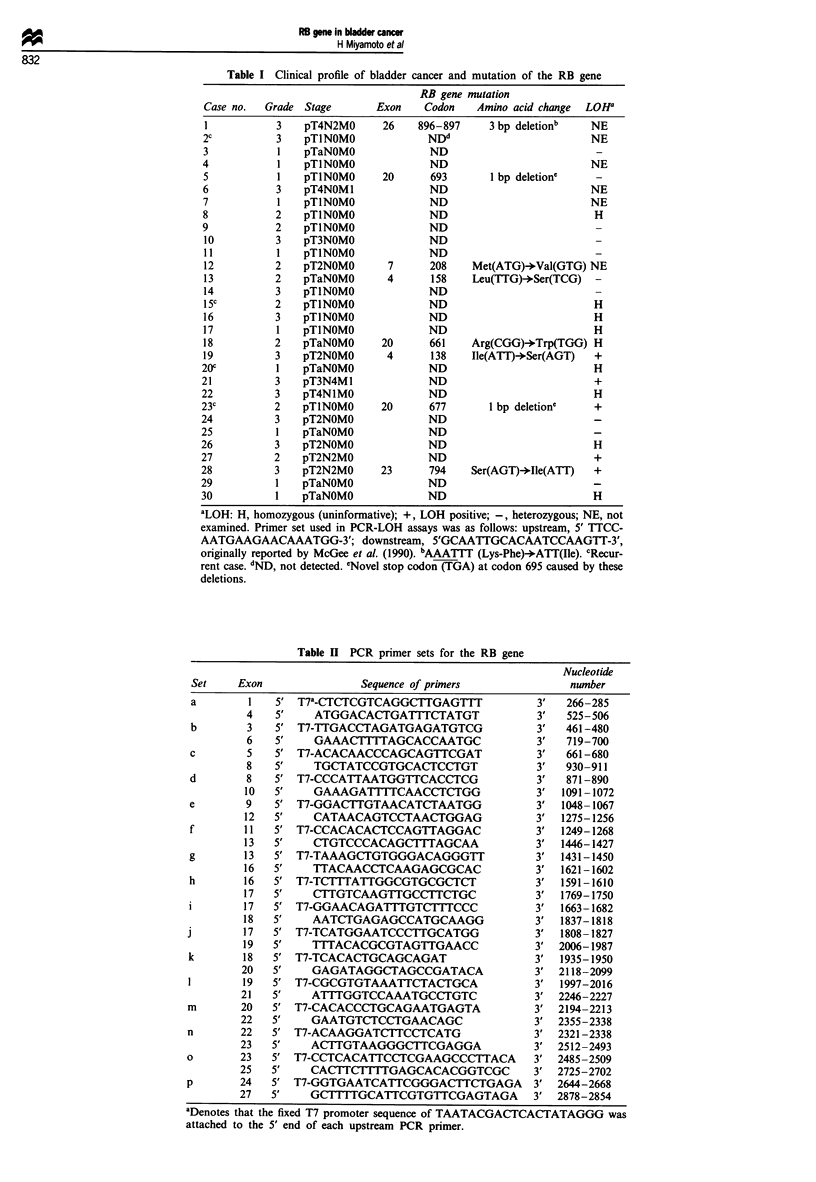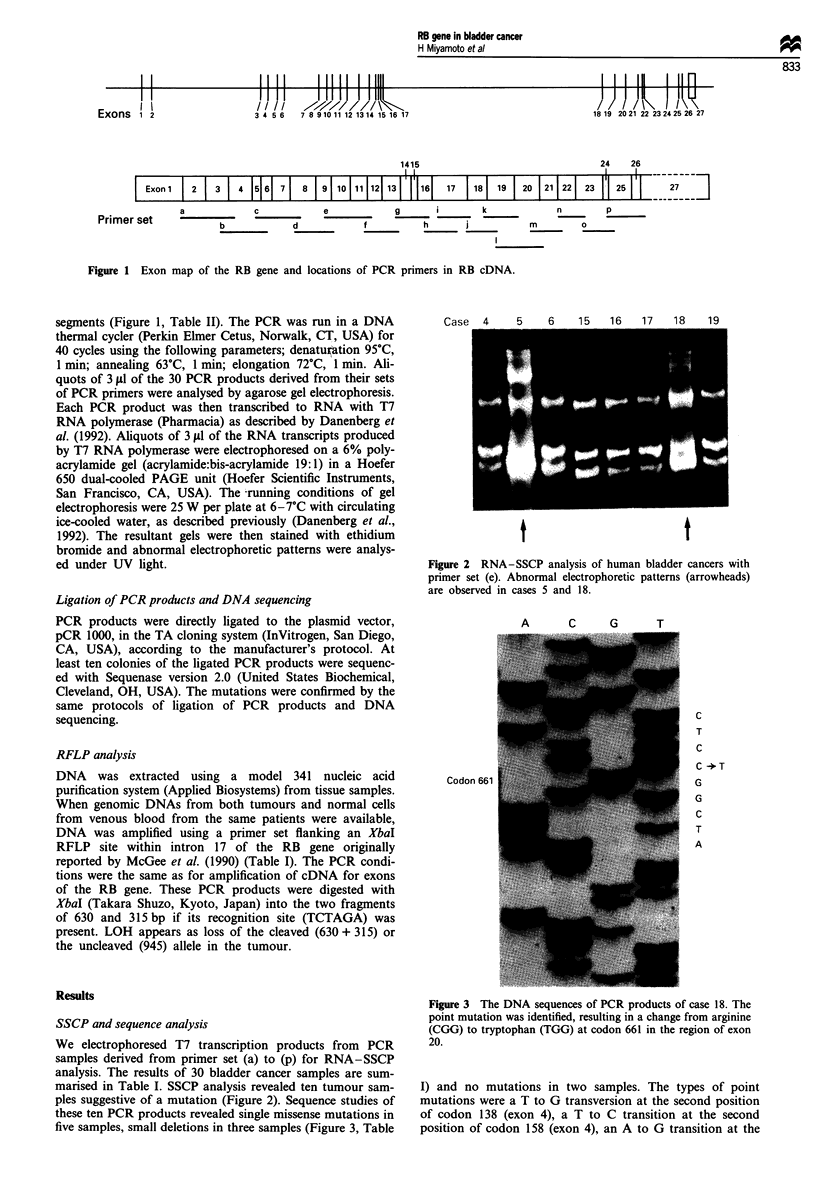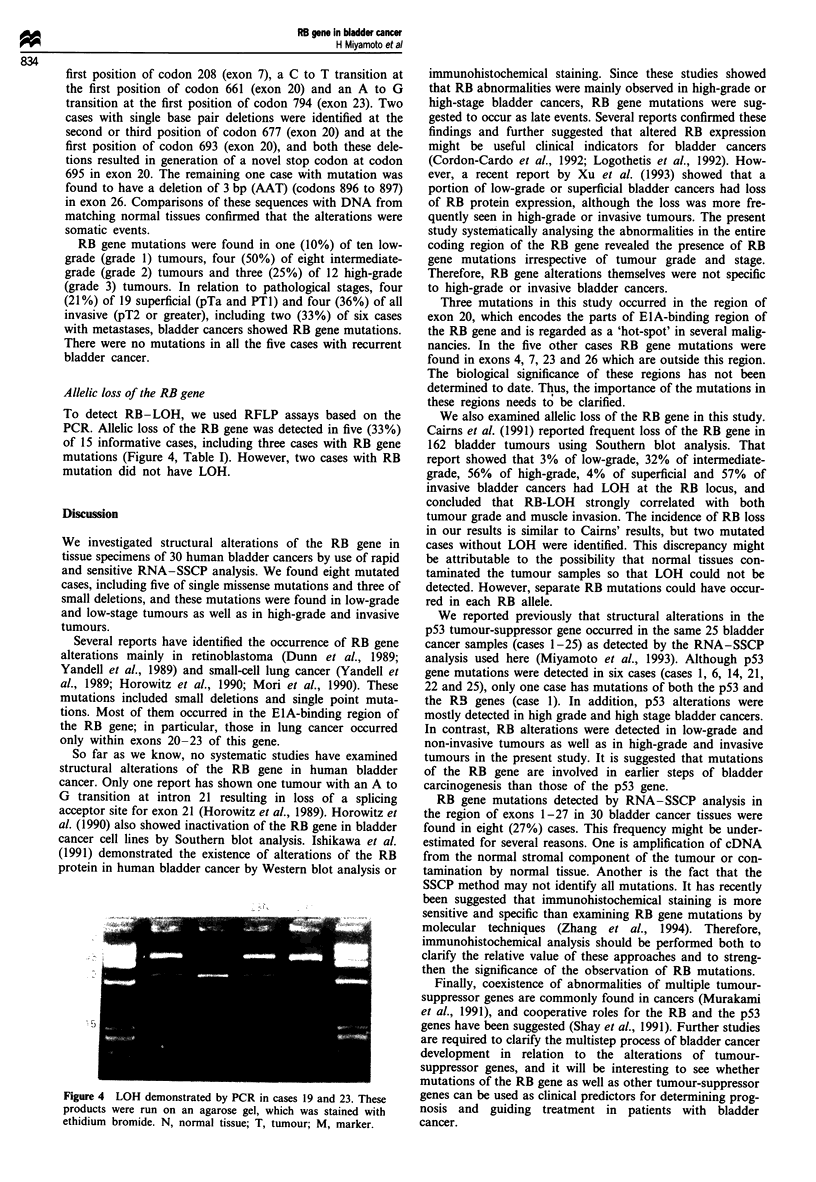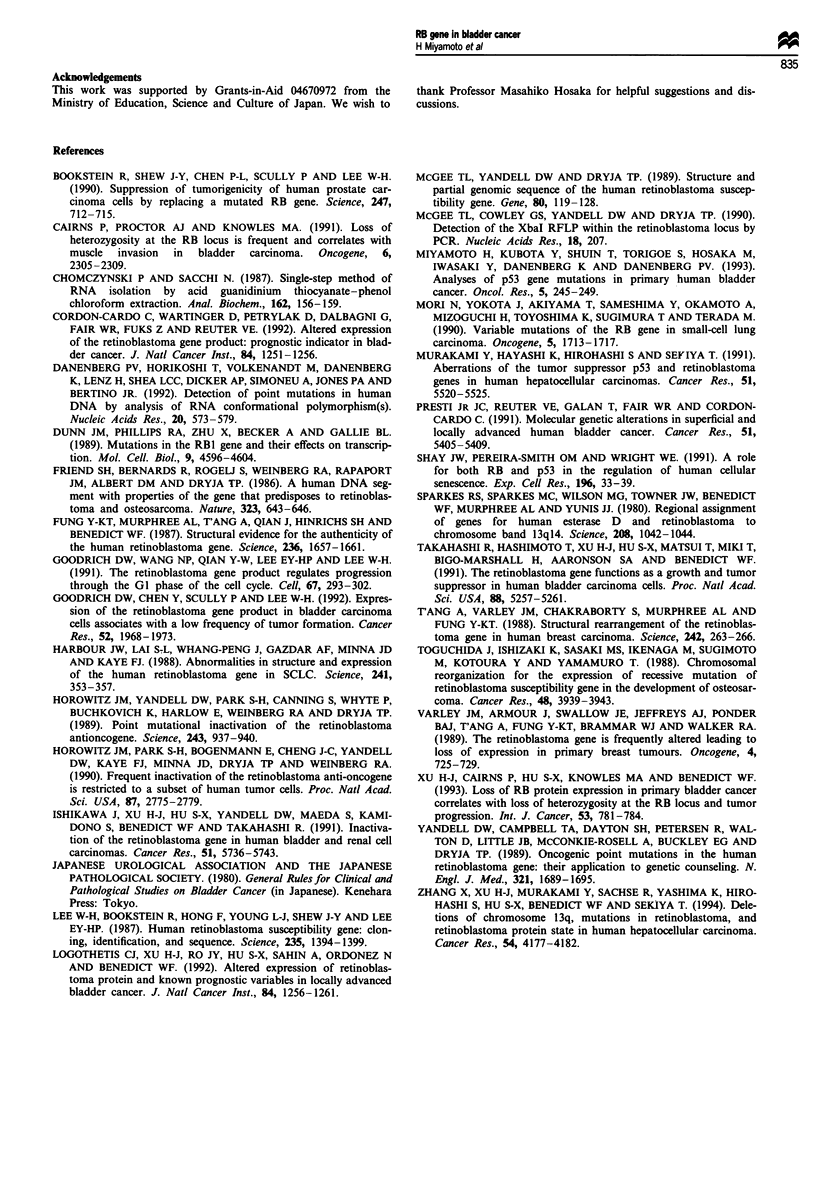# Retinoblastoma gene mutations in primary human bladder cancer.

**DOI:** 10.1038/bjc.1995.160

**Published:** 1995-04

**Authors:** H. Miyamoto, T. Shuin, S. Torigoe, Y. Iwasaki, Y. Kubota

**Affiliations:** Department of Urology, Yokohama City University School of Medicine, Japan.

## Abstract

**Images:**


British Journal of Cancer (1995) 71, 831-835
© 1995 Stockton Press All rights reserved 0007-0920/95 $12.00

### Retinoblastoma gene mutations in primary human bladder cancer

H Miyamoto, T Shuin, S Torigoe, Y Iwasaki and Y Kubota

*Department of Urology, Yokohama City University School of Medicine, 3-9, Fukuura, Kanazawa-ku, Yokohama, 236, Japan.*

**Summary** Inactivation of the retinoblastoma (RB) gene is known to be implicated in the pathogenesis of several types of human cancers. Since structural alterations of the RB gene have not been well examined in human bladder cancer, we looked for mutations in the entire coding region of this gene using polymerase chain reaction (PCR) and single-strand conformational polymorphism analysis of RNA. We also examined allelic loss of the RB gene using PCR-based restriction fragment length polymorphism analysis. Of 30 samples obtained from patients with bladder cancer, eight (27%) were found to have RB gene mutations. DNA sequencing of the PCR products revealed five cases with single point mutations and three cases with small deletions. These mutations included one (10%) of ten low-grade (grade 1) tumours, four (50%) of eight intermediate-grade (grade 2) tumours and three (25%) of 12 high-grade (grade 3) tumours. Likewise, mutations were found in four (21%) of 19 superficial (pTa and pT1) tumours and four (36%) of 11 invasive (pT2 or greater) tumours. In 15 informative cases, loss of heterozygosity at the RB locus was shown in five cases (33%), three cases with RB mutations and two without them. These results suggest that RB gene mutations are involved in low-grade and superficial bladder cancers as well as in high-grade and invasive cancers.

**Keywords:** retinoblastoma gene; bladder cancer; mutation; loss of heterozygosity

The retinoblastoma (RB) susceptibility gene was the first tumour-suppressor gene isolated, is located on human chromosome 13q14 (Sparkes *et al.*, 1980) and consists of 27 exons (McGee *et al.*, 1989). This gene encodes a nuclear phosphoprotein that regulates the cell cycle and forms protein complexes with the adenovirus E1A and SV40 large T oncoproteins (Goodrich *et al.*, 1991). Functional loss of the RB gene is thought to be involved in the initiation and/or progression of many human cancers.

Various studies have revealed that the inactivation of the RB gene has important roles in the development of several types of human tumours, including retinoblastoma (Friend *et al.*, 1986; Fung *et al.*, 1987; Lee *et al.*, 1987), osteosarcoma (Friend *et al.*, 1986; Fung *et al.*, 1987; Toguchida *et al.*, 1988), lung cancer (Harbour *et al.*, 1988; Yandell *et al.*, 1989; Horowitz *et al.*, 1990; Mori *et al.*, 1990), breast cancer (T'Ang *et al.*, 1988; Varley *et al.*, 1989) and prostate cancer (Bookstein *et al.*, 1990). Similarly, several studies have revealed that human bladder cancers have altered or absent RB protein, or loss of heterozygosity (LOH) at the RB locus (Horowitz *et al.*, 1990; Cairns *et al.*, 1991; Ishikawa *et al.*, 1991: Presti *et al.*, 1991; Xu *et al.*, 1993). Takahashi *et al.* (1991) and Goodrich *et al.* (1992) showed that the RB gene transfected into bladder cancer cells suppressed tumorigenicity, suggesting this gene is critical in bladder cancer development. Xu *et al.* (1993) showed alteration of RB protein expression in human bladder cancers by immunohistochemical staining. They also demonstrated that loss of RB protein expression is correlated with LOH at the RB locus. However, structural alterations of the RB gene have not been well examined. In this study, we used the recently developed method of single-strand conformational polymorphism analysis of RNA (RNA-SSCP) (Danenberg *et al.*, 1992) and examined alterations in the entire coding region of the RB gene in 30 primary human bladder cancers of different grades and stages. In addition, we examined RB-LOH using restriction fragment length polymorphism (RFLP) analysis based on the polymerase chain reaction (PCR). RB gene mutations were found in low-grade and non-invasive bladder cancers as well as in high-grade and invasive ones.

Correspondence: Y Kubota
Received 20 July 1994; revised 31 October 1994; accepted 5 December 1994

### Materials and methods

### *Patients and tissues*

Tissue samples of human bladder cancers were obtained from 30 patients [18 males and 12 females, 67.1 ± 14.3 years of age (mean ± s.d.)] by transurethral cold-knife resection, and matching normal tissue samples from heparinised venous blood were collected at Yokohama City University and Yokohama Municipal Citizen's Hospital. Two or three pieces obtained from one tumour in each patient were used for RNA and DNA analyses. The remaining tumours were fixed and haematoxylin and eosin staining was performed for histological diagnosis. All the tumours were histologically diagnosed as transitional cell carcinoma. Twenty-five cases were fresh tumours and five cases were recurrent ones after initial transurethral surgery. None of them had been treated with radiation or anti-cancer drugs before surgery. Detailed clinical and histopathological data on each patient were evaluated at the same institutions according to the General Rule for Clinical and Pathological Studies on Bladder Cancer (Japanese Urological Association and the Japanese Pathological Society, 1980), which adopts the TNM classification system of malignant tumours (Table I). All the samples selected for this study were rapidly frozen in liquid nitrogen and stored at −80°C until RNA or DNA was extracted. Written informed consent was obtained from each patient according to the guidelines of the Human Subjects Reviews Committee.

### *RNA extraction, PCR and RNA-SSCP*

Total cellular RNAs from these tissues were isolated by the acid guanidinium-phenol-chloroform method (Chomczynski and Sacchi, 1987). Isolated RNAs were reverse transcribed to cDNA using random hexamers (Pharmacia, Uppsala, Sweden). The 16 sets of PCR primers shown in Table II and Figure 1 were synthesised according to the RB cDNA sequence described by McGee *et al.* (1989) using an Applied Biosystems model of 392 DNA synthesiser. These primers were designed to cover the coding region from exons 1 to 27 of the RB gene. The T7 RNA polymerase promoter sequence, TAATACGACTCACTATAGGG, was attached to the 5′ end of each upstream PCR primer to produce a single-strand RNA with the same sequence as the sense strand of the cDNA. PCR with each primer set provided PCR products of 150–300 base pairs, covering overlapping

**Table I** Clinical profile of bladder cancer and mutation of the RB gene

| | | | RB gene mutation | | | |
|---|---|---|---|---|---|---|
| *Case no.* | *Grade* | *Stage* | *Exon* | *Codon* | *Amino acid change* | *LOH*[a] |
| 1 | 3 | pT4N2M0 | 26 | 896-897 | 3 bp deletion[b] | NE |
| 2[c] | 3 | pT1N0M0 | | ND[d] | | NE |
| 3 | 1 | pTaN0M0 | | ND | | – |
| 4 | 1 | pT1N0M0 | | ND | | NE |
| 5 | 1 | pT1N0M0 | 20 | 693 | 1 bp deletion[e] | – |
| 6 | 3 | pT4N0M1 | | ND | | NE |
| 7 | 1 | pT1N0M0 | | ND | | NE |
| 8 | 2 | pT1N0M0 | | ND | | H |
| 9 | 2 | pT1N0M0 | | ND | | – |
| 10 | 3 | pT3N0M0 | | ND | | – |
| 11 | 1 | pT1N0M0 | | ND | | – |
| 12 | 2 | pT2N0M0 | 7 | 208 | Met(ATG)→Val(GTG) | NE |
| 13 | 2 | pTaN0M0 | 4 | 158 | Leu(TTG)→Ser(TCG) | – |
| 14 | 3 | pT1N0M0 | | ND | | – |
| 15[c] | 2 | pT1N0M0 | | ND | | H |
| 16 | 3 | pT1N0M0 | | ND | | H |
| 17 | 1 | pT1N0M0 | | ND | | H |
| 18 | 2 | pTaN0M0 | 20 | 661 | Arg(CGG)→Trp(TGG) | H |
| 19 | 3 | pT2N0M0 | 4 | 138 | Ile(ATT)→Ser(AGT) | + |
| 20[c] | 1 | pTaN0M0 | | ND | | H |
| 21 | 3 | pT3N4M1 | | ND | | + |
| 22 | 3 | pT4N1M0 | | ND | | H |
| 23[c] | 2 | pT1N0M0 | 20 | 677 | 1 bp deletion[e] | + |
| 24 | 3 | pT2N0M0 | | ND | | – |
| 25 | 1 | pTaN0M0 | | ND | | – |
| 26 | 3 | pT2N0M0 | | ND | | H |
| 27 | 2 | pT2N2M0 | | ND | | + |
| 28 | 3 | pT2N2M0 | 23 | 794 | Ser(AGT)→Ile(ATT) | + |
| 29 | 1 | pTaN0M0 | | ND | | – |
| 30 | 1 | pTaN0M0 | | ND | | H |

[a]LOH: H, homozygous (uninformative); +, LOH positive; –, heterozygous; NE, not examined. Primer set used in PCR-LOH assays was as follows: upstream, 5′ TTCC-AATGAAGAACAAATGG-3′; downstream, 5′GCAATTGCACAATCCAAGTT-3′, originally reported by McGee *et al.* (1990). [b]AAATTT (Lys-Phe)→ATT(Ile). [c]Recurrent case. [d]ND, not detected. [e]Novel stop codon (TGA) at codon 695 caused by these deletions.

**Table II** PCR primer sets for the RB gene

| *Set* | *Exon* | | *Sequence of primers* | | *Nucleotide number* |
|---|---|---|---|---|---|
| a | 1 | 5′ | T7[a]-CTCTCGTCAGGCTTGAGTTT | 3′ | 266-285 |
| | 4 | 5′ | ATGGACACTGATTTCTATGT | 3′ | 525-506 |
| b | 3 | 5′ | T7-TTGACCTAGATGAGATGTCG | 3′ | 461-480 |
| | 6 | 5′ | GAAACTTTTAGCACCAATGC | 3′ | 719-700 |
| c | 5 | 5′ | T7-ACACAACCCAGCAGTTCGAT | 3′ | 661-680 |
| | 8 | 5′ | TGCTATCCGTGCACTCCTGT | 3′ | 930-911 |
| d | 8 | 5′ | T7-CCCATTAATGGTTCACCTCG | 3′ | 871-890 |
| | 10 | 5′ | GAAAGATTTTCAACCTCTGG | 3′ | 1091-1072 |
| e | 9 | 5′ | T7-GGACTTGTAACATCTAATGG | 3′ | 1048-1067 |
| | 12 | 5′ | CATAACAGTCCTAACTGGAG | 3′ | 1275-1256 |
| f | 11 | 5′ | T7-CCACACACTCCAGTTAGGAC | 3′ | 1249-1268 |
| | 13 | 5′ | CTGTCCCACAGCTTTAGCAA | 3′ | 1446-1427 |
| g | 13 | 5′ | T7-TAAAGCTGTGGGACAGGGTT | 3′ | 1431-1450 |
| | 16 | 5′ | TTACAACCTCAAGAGCGCAC | 3′ | 1621-1602 |
| h | 16 | 5′ | T7-TCTTTATTGGCGTGCGCTCT | 3′ | 1591-1610 |
| | 17 | 5′ | CTTGTCAAGTTGCCTTCTGC | 3′ | 1769-1750 |
| i | 17 | 5′ | T7-GGAACAGATTTGTCTTTCCC | 3′ | 1663-1682 |
| | 18 | 5′ | AATCTGAGAGCCATGCAAGG | 3′ | 1837-1818 |
| j | 17 | 5′ | T7-TCATGGAATCCCTTGCATGG | 3′ | 1808-1827 |
| | 19 | 5′ | TTTACACGCGTAGTTGAACC | 3′ | 2006-1987 |
| k | 18 | 5′ | T7-TCACACTGCAGCAGAT | 3′ | 1935-1950 |
| | 20 | 5′ | GAGATAGGCTAGCCGATACA | 3′ | 2118-2099 |
| l | 19 | 5′ | T7-CGCGTGTAAATTCTACTGCA | 3′ | 1997-2016 |
| | 21 | 5′ | ATTTGGTCCAAATGCCTGTC | 3′ | 2246-2227 |
| m | 20 | 5′ | T7-CACACCCTGCAGAATGAGTA | 3′ | 2194-2213 |
| | 22 | 5′ | GAATGTCTCCTGAACAGC | 3′ | 2355-2338 |
| n | 22 | 5′ | T7-ACAAGGATCTTCCTCATG | 3′ | 2321-2338 |
| | 23 | 5′ | ACTTGTAAGGGCTTCGAGGA | 3′ | 2512-2493 |
| o | 23 | 5′ | T7-CCTCACATTCCTCGAAGCCCTTACA | 3′ | 2485-2509 |
| | 25 | 5′ | CACTTCTTTTGAGCACACGGTCGC | 3′ | 2725-2702 |
| p | 24 | 5′ | T7-GGTGAATCATTCGGGACTTCTGAGA | 3′ | 2644-2668 |
| | 27 | 5′ | GCTTTTGCATTCGTGTTCGAGTAGA | 3′ | 2878-2854 |

[a]Denotes that the fixed T7 promoter sequence of TAATACGACTCACTATAGGG was attached to the 5′ end of each upstream PCR primer.

**Figure 1** Exon map of the RB gene and locations of PCR primers in RB cDNA.

segments (Figure 1, Table II). The PCR was run in a DNA thermal cycler (Perkin Elmer Cetus, Norwalk, CT, USA) for 40 cycles using the following parameters; denaturation 95°C, 1 min; annealing 63°C, 1 min; elongation 72°C, 1 min. Aliquots of 3 µl of the 30 PCR products derived from their sets of PCR primers were analysed by agarose gel electrophoresis. Each PCR product was then transcribed to RNA with T7 RNA polymerase (Pharmacia) as described by Danenberg *et al.* (1992). Aliquots of 3 µl of the RNA transcripts produced by T7 RNA polymerase were electrophoresed on a 6% polyacrylamide gel (acrylamide:bis-acrylamide 19:1) in a Hoefer 650 dual-cooled PAGE unit (Hoefer Scientific Instruments, San Francisco, CA, USA). The running conditions of gel electrophoresis were 25 W per plate at 6–7°C with circulating ice-cooled water, as described previously (Danenberg *et al.*, 1992). The resultant gels were then stained with ethidium bromide and abnormal electrophoretic patterns were analysed under UV light.

### *Ligation of PCR products and DNA sequencing*

PCR products were directly ligated to the plasmid vector, pCR 1000, in the TA cloning system (InVitrogen, San Diego, CA, USA), according to the manufacturer's protocol. At least ten colonies of the ligated PCR products were sequenced with Sequenase version 2.0 (United States Biochemical, Cleveland, OH, USA). The mutations were confirmed by the same protocols of ligation of PCR products and DNA sequencing.

### *RFLP analysis*

DNA was extracted using a model 341 nucleic acid purification system (Applied Biosystems) from tissue samples. When genomic DNAs from both tumours and normal cells from venous blood from the same patients were available, DNA was amplified using a primer set flanking an *Xba*I RFLP site within intron 17 of the RB gene originally reported by McGee *et al.* (1990) (Table I). The PCR conditions were the same as for amplification of cDNA for exons of the RB gene. These PCR products were digested with *Xba*I (Takara Shuzo, Kyoto, Japan) into the two fragments of 630 and 315 bp if its recognition site (TCTAGA) was present. LOH appears as loss of the cleaved (630 + 315) or the uncleaved (945) allele in the tumour.

### Results

### *SSCP and sequence analysis*

We electrophoresed T7 transcription products from PCR samples derived from primer set (a) to (p) for RNA-SSCP analysis. The results of 30 bladder cancer samples are summarised in Table I. SSCP analysis revealed ten tumour samples suggestive of a mutation (Figure 2). Sequence studies of these ten PCR products revealed single missense mutations in five samples, small deletions in three samples (Figure 3, Table I) and no mutations in two samples. The types of point mutations were a T to G transversion at the second position of codon 138 (exon 4), a T to C transition at the second position of codon 158 (exon 4), an A to G transition at the

**Figure 2** RNA–SSCP analysis of human bladder cancers with primer set (e). Abnormal electrophoretic patterns (arrowheads) are observed in cases 5 and 18.

**Figure 3** The DNA sequences of PCR products of case 18. The point mutation was identified, resulting in a change from arginine (CGG) to tryptophan (TGG) at codon 661 in the region of exon 20.

first position of codon 208 (exon 7), a C to T transition at the first position of codon 661 (exon 20) and an A to G transition at the first position of codon 794 (exon 23). Two cases with single base pair deletions were identified at the second or third position of codon 677 (exon 20) and at the first position of codon 693 (exon 20), and both these deletions resulted in generation of a novel stop codon at codon 695 in exon 20. The remaining one case with mutation was found to have a deletion of 3 bp (AAT) (codons 896 to 897) in exon 26. Comparisons of these sequences with DNA from matching normal tissues confirmed that the alterations were somatic events.

RB gene mutations were found in one (10%) of ten low-grade (grade 1) tumours, four (50%) of eight intermediate-grade (grade 2) tumours and three (25%) of 12 high-grade (grade 3) tumours. In relation to pathological stages, four (21%) of 19 superficial (pTa and PT1) and four (36%) of all invasive (pT2 or greater), including two (33%) of six cases with metastases, bladder cancers showed RB gene mutations. There were no mutations in all the five cases with recurrent bladder cancer.

### *Allelic loss of the RB gene*

To detect RB-LOH, we used RFLP assays based on the PCR. Allelic loss of the RB gene was detected in five (33%) of 15 informative cases, including three cases with RB gene mutations (Figure 4, Table I). However, two cases with RB mutation did not have LOH.

### Discussion

We investigated structural alterations of the RB gene in tissue specimens of 30 human bladder cancers by use of rapid and sensitive RNA-SSCP analysis. We found eight mutated cases, including five of single missense mutations and three of small deletions, and these mutations were found in low-grade and low-stage tumours as well as in high-grade and invasive tumours.

Several reports have identified the occurrence of RB gene alterations mainly in retinoblastoma (Dunn *et al.*, 1989; Yandell *et al.*, 1989) and small-cell lung cancer (Yandell *et al.*, 1989; Horowitz *et al.*, 1990; Mori *et al.*, 1990). These mutations included small deletions and single point mutations. Most of them occurred in the E1A-binding region of the RB gene; in particular, those in lung cancer occurred only within exons 20-23 of this gene.

So far as we know, no systematic studies have examined structural alterations of the RB gene in human bladder cancer. Only one report has shown one tumour with an A to G transition at intron 21 resulting in loss of a splicing acceptor site for exon 21 (Horowitz *et al.*, 1989). Horowitz *et al.* (1990) also showed inactivation of the RB gene in bladder cancer cell lines by Southern blot analysis. Ishikawa *et al.* (1991) demonstrated the existence of alterations of the RB protein in human bladder cancer by Western blot analysis or immunohistochemical staining. Since these studies showed that RB abnormalities were mainly observed in high-grade or high-stage bladder cancers, RB gene mutations were suggested to occur as late events. Several reports confirmed these findings and further suggested that altered RB expression might be useful clinical indicators for bladder cancers (Cordon-Cardo *et al.*, 1992; Logothetis *et al.*, 1992). However, a recent report by Xu *et al.* (1993) showed that a portion of low-grade or superficial bladder cancers had loss of RB protein expression, although the loss was more frequently seen in high-grade or invasive tumours. The present study systematically analysing the abnormalities in the entire coding region of the RB gene revealed the presence of RB gene mutations irrespective of tumour grade and stage. Therefore, RB gene alterations themselves were not specific to high-grade or invasive bladder cancers.

**Figure 4** LOH demonstrated by PCR in cases 19 and 23. These products were run on an agarose gel, which was stained with ethidium bromide. N, normal tissue; T, tumour; M, marker.

Three mutations in this study occurred in the region of exon 20, which encodes the parts of E1A-binding region of the RB gene and is regarded as a 'hot-spot' in several malignancies. In the five other cases RB gene mutations were found in exons 4, 7, 23 and 26 which are outside this region. The biological significance of these regions has not been determined to date. Thus, the importance of the mutations in these regions needs to be clarified.

We also examined allelic loss of the RB gene in this study. Cairns *et al.* (1991) reported frequent loss of the RB gene in 162 bladder tumours using Southern blot analysis. That report showed that 3% of low-grade, 32% of intermediate-grade, 56% of high-grade, 4% of superficial and 57% of invasive bladder cancers had LOH at the RB locus, and concluded that RB-LOH strongly correlated with both tumour grade and muscle invasion. The incidence of RB loss in our results is similar to Cairns' results, but two mutated cases without LOH were identified. This discrepancy might be attributable to the possibility that normal tissues contaminated the tumour samples so that LOH could not be detected. However, separate RB mutations could have occurred in each RB allele.

We reported previously that structural alterations in the p53 tumour-suppressor gene occurred in the same 25 bladder cancer samples (cases 1-25) as detected by the RNA-SSCP analysis used here (Miyamoto *et al.*, 1993). Although p53 gene mutations were detected in six cases (cases 1, 6, 14, 21, 22 and 25), only one case has mutations of both the p53 and the RB genes (case 1). In addition, p53 alterations were mostly detected in high grade and high stage bladder cancers. In contrast, RB alterations were detected in low-grade and non-invasive tumours as well as in high-grade and invasive tumours in the present study. It is suggested that mutations of the RB gene are involved in earlier steps of bladder carcinogenesis than those of the p53 gene.

RB gene mutations detected by RNA-SSCP analysis in the region of exons 1-27 in 30 bladder cancer tissues were found in eight (27%) cases. This frequency might be underestimated for several reasons. One is amplification of cDNA from the normal stromal component of the tumour or contamination by normal tissue. Another is the fact that the SSCP method may not identify all mutations. It has recently been suggested that immunohistochemical staining is more sensitive and specific than examining RB gene mutations by molecular techniques (Zhang *et al.*, 1994). Therefore, immunohistochemical analysis should be performed both to clarify the relative value of these approaches and to strengthen the significance of the observation of RB mutations.

Finally, coexistence of abnormalities of multiple tumour-suppressor genes are commonly found in cancers (Murakami *et al.*, 1991), and cooperative roles for the RB and the p53 genes have been suggested (Shay *et al.*, 1991). Further studies are required to clarify the multistep process of bladder cancer development in relation to the alterations of tumour-suppressor genes, and it will be interesting to see whether mutations of the RB gene as well as other tumour-suppressor genes can be used as clinical predictors for determining prognosis and guiding treatment in patients with bladder cancer.

**Acknowledgements**
This work was supported by Grants-in-Aid 04670972 from the Ministry of Education, Science and Culture of Japan. We wish to thank Professor Masahiko Hosaka for helpful suggestions and discussions.

### References

BOOKSTEIN R, SHEW J-Y, CHEN P-L, SCULLY P AND LEE W-H. (1990). Suppression of tumorigenicity of human prostate carcinoma cells by replacing a mutated RB gene. *Science*, **247**, 712-715.

CAIRNS P, PROCTOR AJ AND KNOWLES MA. (1991). Loss of heterozygosity at the RB locus is frequent and correlates with muscle invasion in bladder carcinoma. *Oncogene*, **6**, 2305-2309.

CHOMCZYNSKI P AND SACCHI N. (1987). Single-step method of RNA isolation by acid guanidinium thiocyanate-phenol chloroform extraction. *Anal. Biochem.*, **162**, 156-159.

CORDON-CARDO C, WARTINGER D, PETRYLAK D, DALBAGNI G, FAIR WR, FUKS Z AND REUTER VE. (1992). Altered expression of the retinoblastoma gene product: prognostic indicator in bladder cancer. *J. Natl Cancer Inst.*, **84**, 1251-1256.

DANENBERG PV, HORIKOSHI T, VOLKENANDT M, DANENBERG K, LENZ H, SHEA LCC, DICKER AP, SIMONEU A, JONES PA AND BERTINO JR. (1992). Detection of point mutations in human DNA by analysis of RNA conformational polymorphism(s). *Nucleic Acids Res.*, **20**, 573-579.

DUNN JM, PHILLIPS RA, ZHU X, BECKER A AND GALLIE BL. (1989). Mutations in the RB1 gene and their effects on transcription. *Mol. Cell. Biol.*, **9**, 4596-4604.

FRIEND SH, BERNARDS R, ROGELJ S, WEINBERG RA, RAPAPORT JM, ALBERT DM AND DRYJA TP. (1986). A human DNA segment with properties of the gene that predisposes to retinoblastoma and osteosarcoma. *Nature*, **323**, 643-646.

FUNG Y-KT, MURPHREE AL, T'ANG A, QIAN J, HINRICHS SH AND BENEDICT WF. (1987). Structural evidence for the authenticity of the human retinoblastoma gene. *Science*, **236**, 1657-1661.

GOODRICH DW, WANG NP, QIAN Y-W, LEE EY-HP AND LEE W-H. (1991). The retinoblastoma gene product regulates progression through the G1 phase of the cell cycle. *Cell*, **67**, 293-302.

GOODRICH DW, CHEN Y, SCULLY P AND LEE W-H. (1992). Expression of the retinoblastoma gene product in bladder carcinoma cells associates with a low frequency of tumor formation. *Cancer Res.*, **52**, 1968-1973.

HARBOUR JW, LAI S-L, WHANG-PENG J, GAZDAR AF, MINNA JD AND KAYE FJ. (1988). Abnormalities in structure and expression of the human retinoblastoma gene in SCLC. *Science*, **241**, 353-357.

HOROWITZ JM, YANDELL DW, PARK S-H, CANNING S, WHYTE P, BUCHKOVICH K, HARLOW E, WEINBERG RA AND DRYJA TP. (1989). Point mutational inactivation of the retinoblastoma antioncogene. *Science*, **243**, 937-940.

HOROWITZ JM, PARK S-H, BOGENMANN E, CHENG J-C, YANDELL DW, KAYE FJ, MINNA JD, DRYJA TP AND WEINBERG RA. (1990). Frequent inactivation of the retinoblastoma anti-oncogene is restricted to a subset of human tumor cells. *Proc. Natl Acad. Sci. USA*, **87**, 2775-2779.

ISHIKAWA J, XU H-J, HU S-X, YANDELL DW, MAEDA S, KAMIDONO S, BENEDICT WF AND TAKAHASHI R. (1991). Inactivation of the retinoblastoma gene in human bladder and renal cell carcinomas. *Cancer Res.*, **51**, 5736-5743.

JAPANESE UROLOGICAL ASSOCIATION AND THE JAPANESE PATHOLOGICAL SOCIETY. (1980). *General Rules for Clinical and Pathological Studies on Bladder Cancer* (in Japanese). Kenehara Press: Tokyo.

LEE W-H, BOOKSTEIN R, HONG F, YOUNG L-J, SHEW J-Y AND LEE EY-HP. (1987). Human retinoblastoma susceptibility gene: cloning, identification, and sequence. *Science*, **235**, 1394-1399.

LOGOTHETIS CJ, XU H-J, RO JY, HU S-X, SAHIN A, ORDONEZ N AND BENEDICT WF. (1992). Altered expression of retinoblastoma protein and known prognostic variables in locally advanced bladder cancer. *J. Natl Cancer Inst.*, **84**, 1256-1261.

MCGEE TL, YANDELL DW AND DRYJA TP. (1989). Structure and partial genomic sequence of the human retinoblastoma susceptibility gene. *Gene*, **80**, 119-128.

MCGEE TL, COWLEY GS, YANDELL DW AND DRYJA TP. (1990). Detection of the XbaI RFLP within the retinoblastoma locus by PCR. *Nucleic Acids Res.*, **18**, 207.

MIYAMOTO H, KUBOTA Y, SHUIN T, TORIGOE S, HOSAKA M, IWASAKI Y, DANENBERG K AND DANENBERG PV. (1993). Analyses of p53 gene mutations in primary human bladder cancer. *Oncol. Res.*, **5**, 245-249.

MORI N, YOKOTA J, AKIYAMA T, SAMESHIMA Y, OKAMOTO A, MIZOGUCHI H, TOYOSHIMA K, SUGIMURA T AND TERADA M. (1990). Variable mutations of the RB gene in small-cell lung carcinoma. *Oncogene*, **5**, 1713-1717.

MURAKAMI Y, HAYASHI K, HIROHASHI S AND SEKIYA T. (1991). Aberrations of the tumor suppressor p53 and retinoblastoma genes in human hepatocellular carcinomas. *Cancer Res.*, **51**, 5520-5525.

PRESTI JR JC, REUTER VE, GALAN T, FAIR WR AND CORDON-CARDO C. (1991). Molecular genetic alterations in superficial and locally advanced human bladder cancer. *Cancer Res.*, **51**, 5405-5409.

SHAY JW, PEREIRA-SMITH OM AND WRIGHT WE. (1991). A role for both RB and p53 in the regulation of human cellular senescence. *Exp. Cell Res.*, **196**, 33-39.

SPARKES RS, SPARKES MC, WILSON MG, TOWNER JW, BENEDICT WF, MURPHREE AL AND YUNIS JJ. (1980). Regional assignment of genes for human esterase D and retinoblastoma to chromosome band 13q14. *Science*, **208**, 1042-1044.

TAKAHASHI R, HASHIMOTO T, XU H-J, HU S-X, MATSUI T, MIKI T, BIGO-MARSHALL H, AARONSON SA AND BENEDICT WF. (1991). The retinoblastoma gene functions as a growth and tumor suppressor in human bladder carcinoma cells. *Proc. Natl Acad. Sci. USA*, **88**, 5257-5261.

T'ANG A, VARLEY JM, CHAKRABORTY S, MURPHREE AL AND FUNG Y-KT. (1988). Structural rearrangement of the retinoblastoma gene in human breast carcinoma. *Science*, **242**, 263-266.

TOGUCHIDA J, ISHIZAKI K, SASAKI MS, IKENAGA M, SUGIMOTO M, KOTOURA Y AND YAMAMURO T. (1988). Chromosomal reorganization for the expression of recessive mutation of retinoblastoma susceptibility gene in the development of osteosarcoma. *Cancer Res.*, **48**, 3939-3943.

VARLEY JM, ARMOUR J, SWALLOW JE, JEFFREYS AJ, PONDER BAJ, T'ANG A, FUNG Y-KT, BRAMMAR WJ AND WALKER RA. (1989). The retinoblastoma gene is frequently altered leading to loss of expression in primary breast tumours. *Oncogene*, **4**, 725-729.

XU H-J, CAIRNS P, HU S-X, KNOWLES MA AND BENEDICT WF. (1993). Loss of RB protein expression in primary bladder cancer correlates with loss of heterozygosity at the RB locus and tumor progression. *Int. J. Cancer*, **53**, 781-784.

YANDELL DW, CAMPBELL TA, DAYTON SH, PETERSEN R, WALTON D, LITTLE JB, MCCONKIE-ROSELL A, BUCKLEY EG AND DRYJA TP. (1989). Oncogenic point mutations in the human retinoblastoma gene: their application to genetic counseling. *N. Engl. J. Med.*, **321**, 1689-1695.

ZHANG X, XU H-J, MURAKAMI Y, SACHSE R, YASHIMA K, HIROHASHI S, HU S-X, BENEDICT WF AND SEKIYA T. (1994). Deletions of chromosome 13q, mutations in retinoblastoma, and retinoblastoma protein state in human hepatocellular carcinoma. *Cancer Res.*, **54**, 4177-4182.